# Protein-protein interaction and gene co-expression maps of ARFs and Aux/IAAs in Arabidopsis

**DOI:** 10.3389/fpls.2014.00744

**Published:** 2014-12-23

**Authors:** Sarbottam Piya, Sandesh K. Shrestha, Brad Binder, C. Neal Stewart, Tarek Hewezi

**Affiliations:** ^1^Department of Plant Sciences, University of TennesseeKnoxville, TN, USA; ^2^Department of Entomology and Plant Pathology, University of TennesseeKnoxville, TN, USA; ^3^Department of Biochemistry, Cellular, and Molecular Biology, University of TennesseeKnoxville, TN, USA

**Keywords:** Arabidopsis, auxin, auxin/indole-3-acetic acid proteins, auxin response transcription factors, protein-protein interactions, co-expression network

## Abstract

The phytohormone auxin regulates nearly all aspects of plant growth and development. Based on the current model in *Arabidopsis thaliana*, Auxin/indole-3-acetic acid (Aux/IAA) proteins repress auxin-inducible genes by inhibiting auxin response transcription factors (ARFs). Experimental evidence suggests that heterodimerization between Aux/IAA and ARF proteins are related to their unique biological functions. The objective of this study was to generate the Aux/IAA-ARF protein-protein interaction map using full length sequences and locate the interacting protein pairs to specific gene co-expression networks in order to define tissue-specific responses of the Aux/IAA-ARF interactome. Pairwise interactions between 19 ARFs and 29 Aux/IAAs resulted in the identification of 213 specific interactions of which 79 interactions were previously unknown. The incorporation of co-expression profiles with protein-protein interaction data revealed a strong correlation of gene co-expression for 70% of the ARF-Aux/IAA interacting pairs in at least one tissue/organ, indicative of the biological significance of these interactions. Importantly, ARF4-8 and 19, which were found to interact with almost all Aux-Aux/IAA showed broad co-expression relationships with *Aux/IAA* genes, thus, formed the central hubs of the co-expression network. Our analyses provide new insights into the biological significance of ARF-Aux/IAA associations in the morphogenesis and development of various plant tissues and organs.

## Introduction

The plant hormone auxin (indole-3-acetic acid; IAA), regulates a wide range of developmental and physiological processes in plants including for example, apical dominance, root development, vascular differentiation, shoot elongation, and embryo patterning (Benjamins and Scheres, 2008; Zhao, 2010). Also, auxin regulates various cellular processes that are associated with plant responses to biotic and abiotic stresses (Kazan and Manners, 2009).

Characterization of plant responses to IAA treatments led to the identification of various classes of early auxin-responsive genes. Members of the *Aux/IAA* gene family were among the first auxin-regulated genes to be identified. In Arabidopsis, the *Aux/IAA* gene family comprises 29 members and encodes short-lived nuclear proteins. The hallmark characteristic of Aux/IAA proteins is the presence of four highly conserved domains (domains I–IV), which underlie the functional properties of these proteins. Domain I mediates the transcriptional repression of the proteins, whereas domain II mediates protein degradation (Chapman and Estelle, 2009). Domains III and IV are responsible for homo- and hetero-dimerization with other Aux/IAA proteins as well as heterodimerization with the auxin response factors (ARFs) (Chapman and Estelle, 2009). In Arabidopsis, ARFs are encoded by a large gene family containing 22 members. These transcription factors bind specifically to auxin-responsive *cis*-acting elements that are frequently found in the promoters of early auxin-responsive genes. Aux/IAA proteins negatively regulate the abundance of ARFs, and subsequently the expression of auxin-responsive genes. Extensive molecular and biochemical analyses revealed the mechanism of this regulation (Chapman and Estelle, 2009). More specifically, in the presence of auxin, the degradation of the Aux/IAA proteins is enhanced, thus alleviating the repression of ARF activity and allowing them to drive the transcription of auxin-responsive genes. By contrast, in the absence of auxin, Aux/IAA protein levels increase and they bind to AFRs to inhibit their function. Based on this model, auxin signaling involves TRANSPORT INHIBITOR RESPONSE1/AUXIN-BINDING F-BOX PROTEIN (TIR1/AFB) auxin receptors, Aux/IAA inhibitors, and ARF *cis*-acting transcription factors regulating the expression of auxin-responsive genes. Because these proteins are encoded by multigene families in Arabidopsis (5 TIR/AFB, 29 Aux/s and 22 ARFs) there are opportunities for numerous combinatorial interactions among these proteins to mediate specific responses.

Experimental evidence suggests that heterodimerization between Aux/IAA and ARF proteins are important to define their unique biological functions (Weijers et al., 2005). For example, ARF7 was found to be regulated by Aux/IAA3 in roots and by Aux/IAA19 in hypocotyls (Tatematsu et al., 2004), suggesting that the activities of ARFs could be regulated by different Aux/IAA proteins in a tissue-dependent fashion. Thus, it is possible that the formation of a wide range of dimer combinations among and between these two gene family proteins represents the mechanisms by which ARF transcription factors regulate diverse cellular processes. Several studies have reported on combinatorial protein-protein interactions between ARFs and Aux/IAAs (Fukaki et al., 2005; Weijers et al., 2005; Uehara et al., 2008; Li et al., 2011; Vernoux et al., 2011; Arase et al., 2012). However, most of these studies used partial ARF sequences containing the C-terminal protein-protein interaction domain (CTD) and discrepancies in protein-protein interactions between Aux/IAA proteins and that of the full-length or truncated ARFs are frequently found. For example, Aux/IAA17 was found to interact strongly with the full-length of ARF1 (Ouellet et al., 2001) but no interaction was detected when a truncated version of ARF1 containing the CTD was used (Tiwari et al., 2003; Vernoux et al., 2011). Similarly, differences in interaction intensity between rice Aux/IAAs and intact or truncated versions of ARFs were observed (Shen et al., 2010).

The global protein-protein interaction network of a specific gene family provides information of all physical associations that can occur among family members. However, weighing the biological significance of such an interactome is a real challenge because of tissue specificities and the dynamic nature of protein-protein interactions. Global gene co-expression analysis has recently emerged as a powerful approach to identify the tissues and the conditions in which important interactions occur. This is based on the idea that proteins can physically interact in particular cell types or tissues only if their genes are co-expressed in these cell types or tissues. The integration of global gene expression data with a protein interaction network has been used to determine the cellular conditions and tissues specificity of protein interaction network of human proteins (Bossi and Lehner, 2009), and Arabidopsis MADS Box transcription factors (De Folter et al., 2005), cell cycle proteins (Boruc et al., 2010), and G-proteins (Klopffleisch et al., 2011).

In this study, we generated the protein–protein interaction map of Arabidopsis ARF and Aux/IAA proteins using full-length sequences in yeast two-hybrid assays. We identified 213 specific interactions between ARFs and Aux/IAAs in which 79 interactions have not been reported previously. In addition, we integrated the global gene co-expression profile with the protein interaction map and identified tissue-specificity for the majority of the ARF-Aux/IAAs interactions.

## Materials and methods

### Plasmid construction

Full-length coding sequences for 19 *ARFs* and 29 *Aux/IAAs* were obtained from Arabidopsis Biological Resource Center or isolated from cDNA. The coding sequences of these *ARF* and *Aux/IAA* genes were PCR amplified using forward and reverse primers containing specific restriction enzyme sites (Supplemental Table 1). PCR amplification was performed using PrimeSTAR GXL DNA polymerase (Takara Bio) following manufacturers' instructions. PCR products of *ARFs* were digested, purified and fused to the GAL4 DNA binding domain of the pGBKT7 vector (Clontech) to generate pGBKT7-ARFs. Similarly, PCR products of *Aux/IAAs* were digested, purified and fused to the GAL4 DNA activation domain of pGADT7 vector (Clontech) to generate pGADT7-Aux/IAAs. All constructs were verified by sequencing.

### Yeast two-hybrid assays

*Saccharomyces cerevisiae* strain AH109 was co-transformed with 551 pairs of pGBKT7-ARF and pGADT7-Aux/IAA vectors and yeast cells containing both vectors were selected using SD/-Leu/-Trp medium. The interactions between ARFs and Aux/IAA were assayed by plating the transformed cells onto the stringent SD/-Ade-His-Leu-Trp selective medium using at least 10 independent colonies. Serial dilutions of yeast co-transformed cells were used to measure the strength of the interaction.

### Bimolecular fluorescence complementation (BiFC) assays

The coding sequence of *ARF5, 6*, and *19* were PCR amplified using forward and reverse primers containing restriction enzyme sites (Supplemental Table 1). After digestion, Purified PCR products were cloned into pSAT4-cEYFP-C1-B to generate cEYFP-ARF5, 6, and 19 fusions. Likewise, *Aux/IAA5, 6, 17, 32*, and *34* were cloned into pSAT4-nEYFP-C1 to generate nEYFP-*Aux/IAA5, 6, 17, 32*, and *34* fusions. All constructs were verified by sequencing. Ten combinations of cEYFP and nEYFP fusions, in addition to controls, were co-expressed in onion (*Allium cepa*) epidermal cells using particle bombardment as previously described by Hewezi et al. (2008). Co-transformed tissues were incubated at 25°C in dark for 16–24 h before being assayed for YFP activity. Bright field and fluorescent images were observed using EVOS® FL Auto Cell Imaging System (Life Technologies).

### Gene co-expression network analysis

Raw RNA seq data for 65 samples including root, shoot apical meristem, seedlings, hypocotyls, leaves, floral buds, flowers, embryos, and whole plants were downloaded from NCBI Sequence Read Archive (SRA) (Leinonen et al, 2011). The SRA files were converted to FASTQ file using fastq-dump of SRA Toolkit (http://www.ncbi.nlm.nih.gov/Traces/sra/sra.cgi?view=toolkit_doc&f=fastq-dump). Quality assessment of sequence reads was performed using FastQC (http://www.bioinformatics.babraham.ac.uk/projects/fastqc/). Quality filtering and trimming were conducted using FASTX-Toolkit (http://hannonlab.cshl.edu/fastx_toolkit/). After quality filtering and trimming, each sequence read was aligned to the TAIR10 *Arabidopsis thaliana* reference genome (Swarbreck et al., 2008) using TopHat v2.0.11 (Trapnell et al., 2009) (Supplemental Table 2). The output from TopHat (bam file) was used to quantify gene expression level as FPKM (fragments per kilobase of transcript per million mapped reads) using Cufflink v2.2.1 (Trapnell et al., 2010). Output from cufflink was filtered to extract the expression value for *ARF* and *Aux/IAA* genes using AWK command (Supplemental Table 3). FPKM value of 2 was used as a threshold for expressed genes, and hence, only those genes having FPKM values more than two in at least one tissue were included in the gene co-expression analysis. To determine the tissues in which *ARF-Aux/IAA* pairs are co-expressed, we computed the Z-score for each of the FPKM values (Supplemental Table 4). The Z-score values were averaged across different samples of a given tissue and positive values of Z-score indicate high expression (Supplemental Table 4). *ARF-Aux/IAA* combinations are considered co-expressed in a tissue only if Z-score for both genes in this tissue is positive with a *P* value less than 0.05. A heatmap of all co-expressed *ARF-Aux/IAA* pairs in various tissues was constructed using sample contribution score (Supplemental Table 5) in Multi Experiment Viewer (http://www.tm4.org/mev.html). Sample contribution scores were calculated by multiplying Z-score of *ARFs* and *Aux/IAAs* for each tissue as described in CoexViewer available at ATTED-II database (Obayashi et al., 2014). Positive values of sample contribution score resulting from negative Z-scores of both *ARFs* and *Aux/IAAs* was made negative. Cytoscape (Shannon et al., 2003) was used to integrate gene co-expression data with protein-protein interaction data.

### Phylogenetic analysis

Protein sequences of all ARFs and Aux/IAAs were downloaded from the The Arabidopsis Information Resource (TAIR) website. Sequences were aligned using ClustalX (Jeanmougin et al., 1998) and neighbor-joining tree was constructed using MEGA6 (Tamura et al., 2013) with default settings.

## Results

### Construction of comprehensive interaction map of ARFs and Aux/IAA

**Figure 1:**
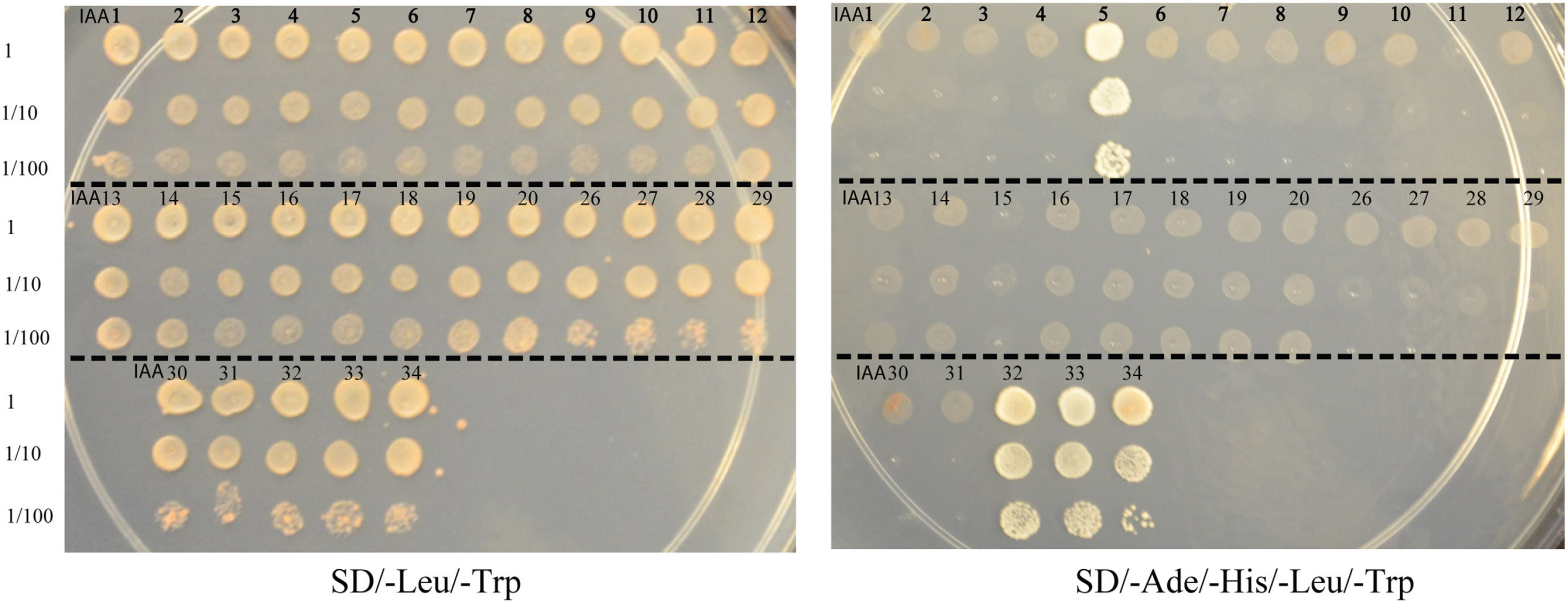
**Yeast two-hybrid interaction between ARF10 and various Aux/IAAs**. Yeast strain AH109 was co-transformed with *ARF10* bait vector in combination with 29 *Aux/IAA* prey vectors. Protein-protein interactions between ARF10 and each of Aux/IAA5, Aux/IAA32, Aux/IAA33 and Aux/IAA34 were visualized by differential growth on the SD/-Leu/-Trp non-selective medium (left) and on the SD/-Ade/-His/-Leu/-Trp selective medium (right).

**Figure 2:**
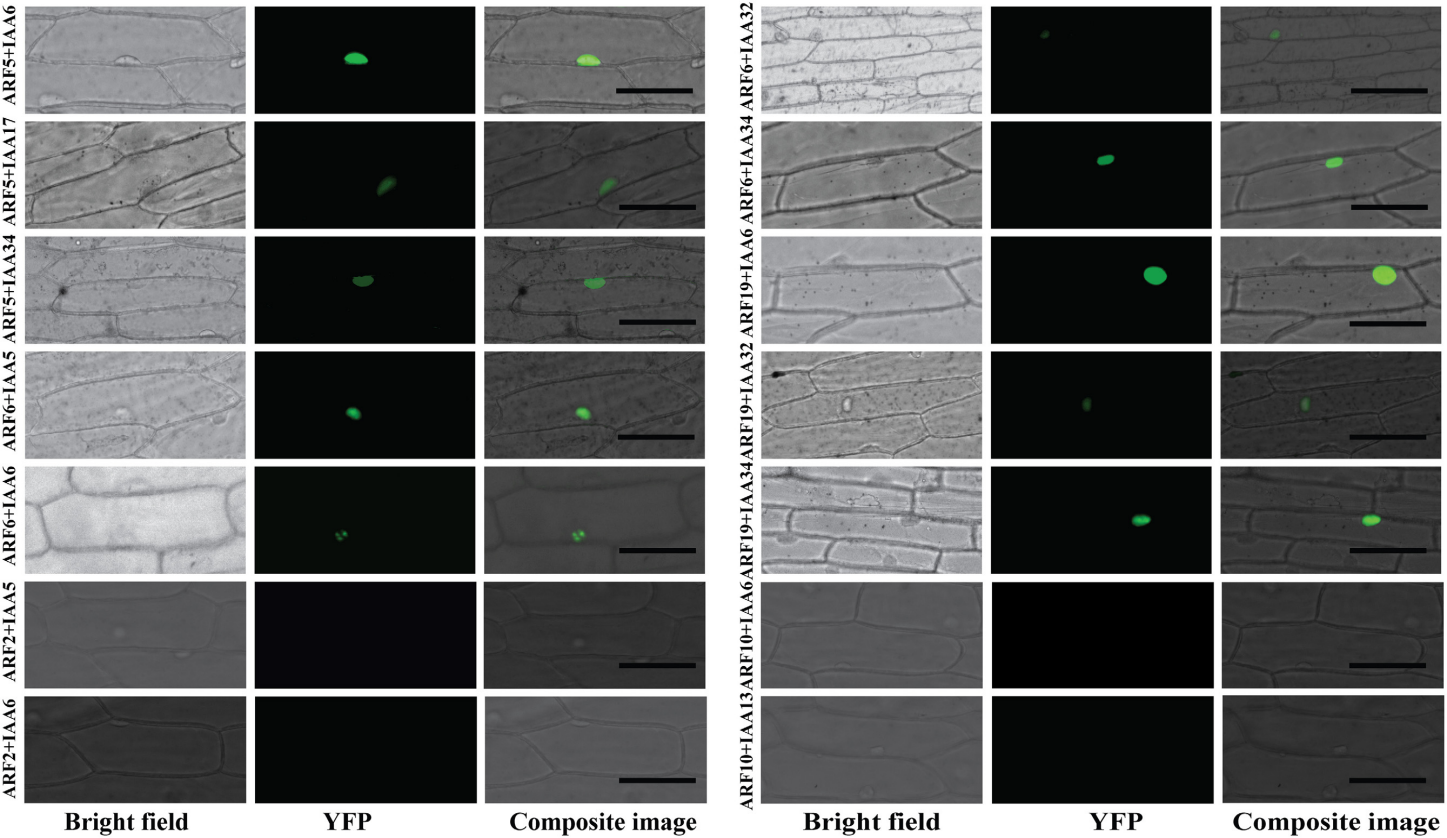
**BiFC visualization of the ARF-Aux/IAA interactions**. Onion epidermal cells were co-transformed with ten different combinations of constructs expressing cEYFP-ARF5, 6, and 19 fusions and nEYFP-Aux/IAA5, 6, 17, 32, and 34 fusions. Bright field, YFP and composite images were taken 16–24 h after bombardment. Four combinations of non-interacting ARF-Aux/IAA pairs in yeast (ARF2-Aux/IAA5, ARF2-Aux/IAA6, ARF10-Aux/IAA6 and ARF10-Aux/IAA13) were used as negative control and showed no YFP signal. Bar = 100 μM.

To generate a comprehensive protein-protein interaction map between ARF and Aux/IAA proteins, yeast co-transformation assays were performed between 19 ARFs and 29 Aux/IAAs. The full-length coding sequences of 19 ARFs (ARF1-13 and ARF16-20 and ARF22) were cloned in a bait vector and full-length coding sequences of 29 Aux/IAAs (Aux/IAA1-20 and Aux/IAA26-34) were cloned in a prey vector. Yeast cells were co-transformed with 551 pairs of bait and prey vectors and potential interactions were visualized by differential growth on the non-selective synthetic dropout (SD) medium (SD/-Leu/-Trp) and on the selective medium (SD/-Leu/-Trp/-His/-Ade). An example of the interaction between ARF10 and Aux/IAAs is provided in Figure 1. Of the 551 interactions tested, 213 interactions between ARFs and Aux/IAAs were detected. To confirm the protein-protein interactions *in planta*, bimolecular fluorescence complementation (BiFC) assays (Citovsky et al., 2006) were performed with ARFs and Aux/IAAs that displayed weak-to-strong interactions in yeast. Coding sequences of ARF5, 6, and 19 were fused to the N-terminal half of a yellow fluorescent protein gene (*nEYFP*), while Aux/IAA5, 6, 17, 32, and 34 were fused to the C-terminal half of a yellow fluorescent protein gene (*cEYFP*). Ten different combinations between nEYFP and cEYFP fusions were co-expressed in onion epidermal cells. All ARF-Aux/IAA interaction combinations, including those showing weak interaction in yeast (ARF6-Aux/IAA32 and ARF19-Aux/IAA34), reconstituted the fluorescent YFP in the nucleus of transformed cells (Figure 2), validating our yeast co-transformation data. Onion epidermal cells co-transformed with 4 non-interacting pairs yielded no YFP fluorescence.

**Figure 3:**
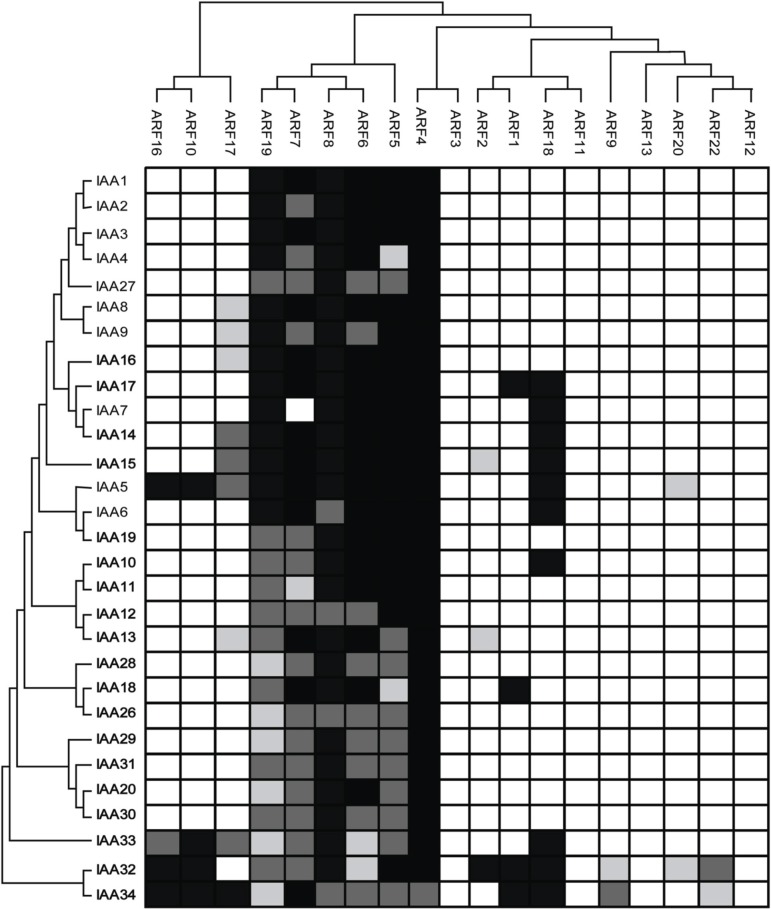
**Protein-protein interaction map of Arabidopsis ARF and Aux/IAA proteins**. ARFs (top) and Aux/IAAs (left) are arranged according to their sequence similarity. Empty boxes indicate no interaction while gray and black boxes indicate weak and strong interaction, respectively.

It is interesting to note that all ARFs that function as activators (ARF5, ARF6, ARF7, ARF8, and ARF19) were found to interact with all Aux/IAA proteins except ARF7 with Aux/IAA7 (Figure 3). In contrast, ARFs that function as repressors showed interactions with certain Aux/IAAs. One exception is ARF4, which interacted strongly with all Aux/IAA proteins. One remarkable finding is that ARFs that functioned as repressors dimerized preferentially with Aux/IAA32 and Aux/IAA34 (Figure 3). For example, out of the 14 ARFs we included in our analysis that functioned as repressors 9 and 8 ARFs were found to interact with Aux/IAA32 and Aux/IAA34, respectively (Figure 3). Of these ARFs, ARF1, 10, 16, and 18 showed strong interactions with both Aux/IAA32 and 34. No interactions were detected for ARF3, ARF11, ARF12 and ARF13. ARF3 and ARF13 do not contain the C-terminal protein-protein interaction domain (CTD), which mediates the interaction with Aux/IAAs through binding to motif III and IV found in Aux/IAAs. ARF17 does not contain the CTD but was found to interact with 9 Aux/IAA proteins including Aux/IAA5, 8, 9, 13–16, 33, and 34 (Figure 3).

### Phylogenetically-related ARFs exhibited various interactions with Aux/IAA proteins

We tested whether phylogenetically-related ARF and Aux/IAA proteins would have similar protein-protein interaction patterns. In general, we found that phylogenetically-related ARF and Aux/IAA proteins formed similar protein-protein interactomes. For example, ARF5-8 and ARF19, which are phylogenetically-related, interacted with almost all Aux/IAA proteins. Similarly, ARF10 and 16, which clustered together, were found to interact with the same Aux/IAA proteins including Aux/IAA5, and Aux/IAA32-34 (Figure 3). In contrast, the closely-rated ARF3 and 4 showed distinct interaction patterns. While ARF4 interacted strongly with all Aux/IAA protein, none of the Aux/IAA proteins was detected as ARF3 interactor, perhaps owing to the absence of the CTD in ARF3. Likewise, ARF11 and 18 showed different interaction patterns despite the fact that they are phylogenetically-related.

### Co-expression analysis of interacting ARF-Aux/IAA proteins

**Figure 4:**
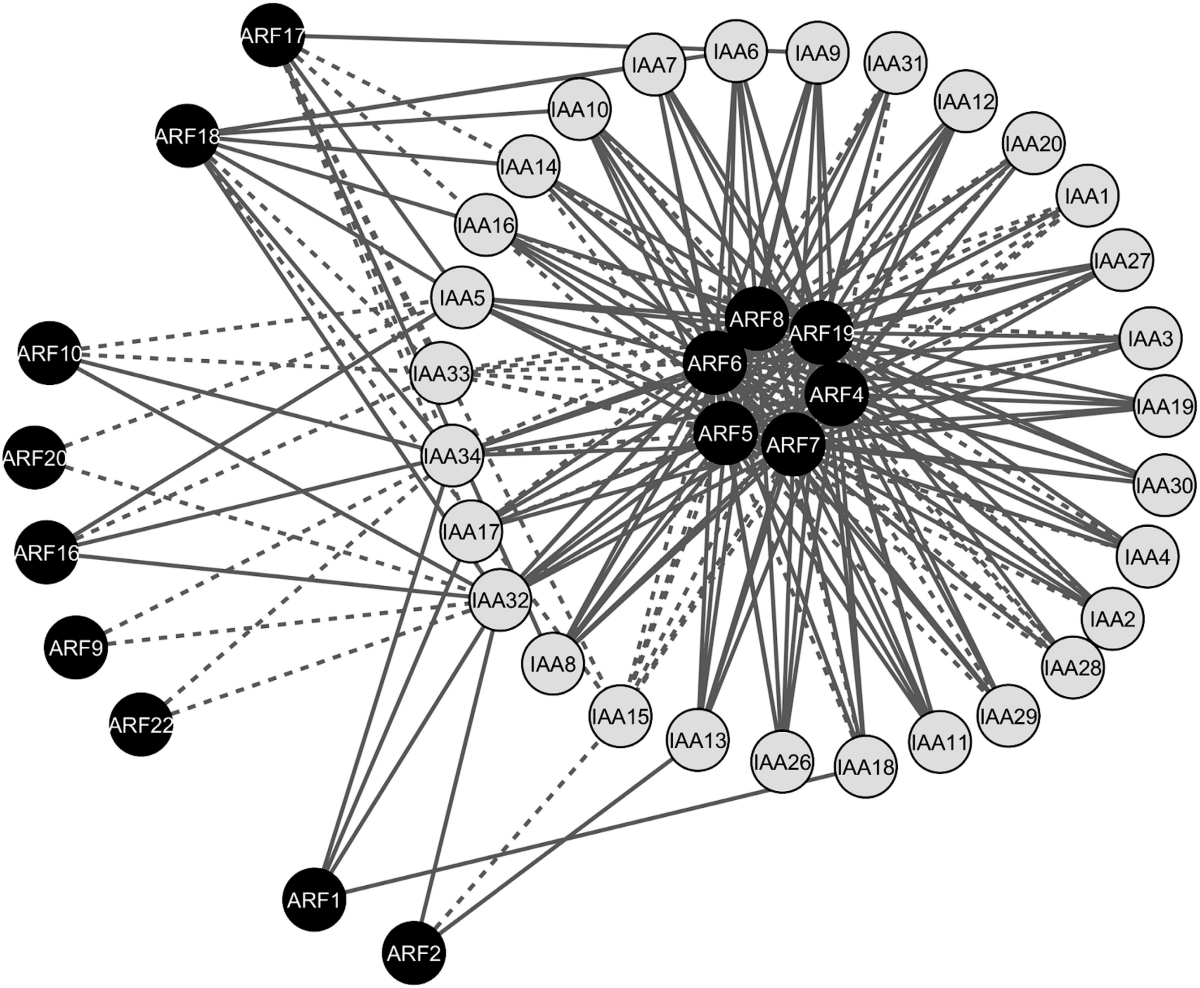
**Gene co-expression network of the interacting ARF-Aux/IAA proteins**. The network contained 44 nodes (15 ARFs and 29 Aux/IAAs) and 213 edges (interacting combinations). Continuous edges indicate protein pairs with significantly correlated expression profiles in at least one tissue, whereas dotted edges indicate protein pairs without significantly correlated expression profiles.

Auxin-specific response in plant tissues is defined by specific ARF-Aux/IAA pairs that co-express in these tissues (Weijers et al., 2005). Therefore, interacting ARF-Aux/IAA pairs that co-express in particular tissues are the potential combinations that facilitate the conversion of auxin signal into specific responses during morphogenesis. To test the biological significance of the physical ARF-Aux/IAA associations, we integrated the protein interaction map with the co-expression map. We analyzed gene co-expression profiles of *ARF* and *Aux/IAA* genes in different Arabidopsis tissues/organs using 65 different RNAseq datasets from the SRA (Leinonen et al, 2011). Pair-wise gene co-expression values of genes encoding ARFs and Aux/IAAs in various tissues and organs were used to generate the co-expression network of the ARF-Aux/IAA interacting proteins shown in Figure 4. The network included 44 nodes (15 ARFs and 29 Aux/IAA) and 213 edges (interacting combinations). Out of the 213 interacting combinations, 149 combinations (70%) had co-expression patterns in at least one tissue and were represented by continuous edges (Figure 4). The remaining 64 interacting combinations did not show significant co-expression relationships, represented by dotted edges (Figure 4). ARF4-8 and 19, which were found to interact with almost all Aux/IAAs, showed broad co-expression relationships with *Aux/IAA* genes, and thus constituted the central hubs of the map. Notably, all the interacting combinations of ARF1, 2, and 16 showed significant co-expression correlations. In contrast, ARF20, ARF22 and Aux/IAA33 did not show any co-expression association in the tissues included in our analysis.

**Figure 5:**
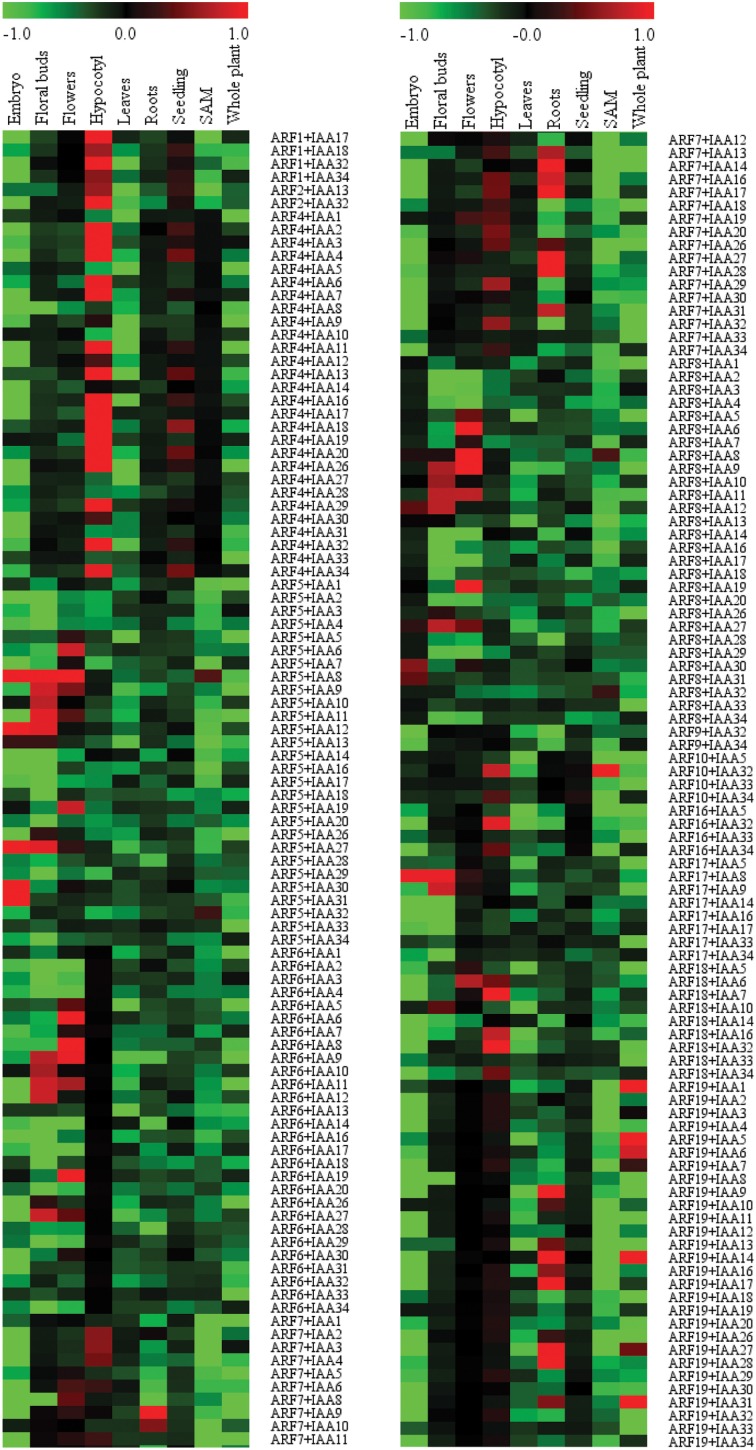
**Heatmap demonstrating gene co-expression patterns of the interacting ARF-Aux/IAA proteins in various Arabidopsis tissues and organs**. Sample contribution scores were calculated for each ARF-Aux/IAA interacting combinations (Supplemental Table 5) and used to construct the heatmap using the Multi Experiment Viewer (http://www.tm4.org/mev.html). Red color represents pairs with highly correlated gene co-expression profiles and green represents highly anti-correlated pairs.

In order to map the co-expression events of the interacting ARF-Aux/IAA proteins to specific tissues or organs, the co-expression between pairs was determined using Z score as described in the Material and Methods. We set the “fragments per kilobase of transcript per million mapped reads” (FPKM) value of 2.0 as the threshold for expressed genes. As a result, *ARF12*-*14, ARF20, ARF22* and *Aux/IAA15*, which have FPKM value less than 2 were considered as non-expressed in the samples included and hence, were not included in the co-expression analysis (Supplemental Table 3). Also, *IAA33* and *IAA34* showed very low expression levels across all tissues except in the roots and seedlings, respectively (Supplemental Table 3). The co-expression profiles of *ARFs* and *Aux/IAAs* in embryos, floral buds, flowers, hypocotyls, leaves, roots, shoot apical meristems, seedlings, and whole plant are presented as a heatmap in Figure 5. It is very interesting to note that we observed general trends of co-expression specificity in which the majority of the *ARF*/*Aux/IAA* pairs are expressed in only one tissue/organ. In few cases, the co-expression associations of the pairs were found in two and to a much lower extent in three tissues, these occurred in tissues with related functions such as flowers, floral buds and embryos. For example, *ARF5-Aux/IAA12, ARF5-Aux/IAA27*, and *ARF17-Aux/IAA8* were co-expressed in floral buds and embryos. Similarly, *ARF6-Aux/IAA9, ARF6-Aux/IAA11, ARF8-Aux/IAA9*, and *ARF8/Aux/IAA11* were co-expressed in flowers and floral buds. It is also interesting to note that in each tissue, several co-expression events occurred in which only a few ARFs were observed. For example, the 16 co-expression events detected in roots were contributed by *ARF7* and *ARF19*. In other tissues, the number of co-expression events was very limited. For example, in SAM only 5 co-expression events between *ARFs* and *Aux/IAAs* were detected (*ARF5-Aux/IAA8, ARF5-Aux/IAA32, ARF8-Aux/IAA8, ARF8-Aux/IAA32*, and *ARF10-Aux/IAA32*).

## Discussion

Models for auxin signal transduction pathway have positioned ARF and Aux/IAA proteins centrally in the network in which auxin signals are converted into specific physiological responses (Muday et al., 2012). ARFs contain N-terminal DNA binding domain that binds to the TGTCTC *cis* regulatory element in the promoters of auxin-response genes, a middle region (MR) that functions as activator or repressor, and frequently a CTD involved in protein-protein interactions (Guilfoyle and Hagen, 2007). Likewise, Aux/IAAs contain an N-terminal repression domain (domain I), a degradation domain (domain II) that facilitates degradation of Aux/IAAs through ubiquitin–proteasome pathway in response to auxin, and protein-protein interaction domains (domain III and IV), which resemble CTD of the ARFs (Guilfoyle and Hagen, 2012). The CTD of ARFs and domains III and IV of Aux/IAAs mediate ARF-Aux/IAA heterodimerization (Weijers et al., 2005). Because ARF and Aux/IAA proteins are encoded by multigene families, there are opportunities for many combinatorial interactions between these proteins, which apparently are necessary to mediate auxin-specific responses in various developmental and physiological contexts. Studies of protein-protein interactions between Arabidopsis ARFs and Aux/IAAs have been conducted using truncated ARFs with only CTDs (Tiwari et al., 2003; Tatematsu et al., 2004; Szemenyei et al., 2008; Vernoux et al., 2011) with the assumption that the ARF protein domains other than CTD have no role in facilitating ARF-Aux/IAA interactions. Although the interaction between these two gene families are mediated through the CTD, using truncated proteins might not be physiologically relevant in that the result could be instable or misfolded protein domains or fragments that could impact the interaction outcomes.

In this study, we identified 213 specific interactions between 19 ARFs and 29 Aux/IAAs using yeast two-hybrid assays. A subset of 10 interacting combinations in yeast was also validated *in planta* using BiFC, indicating that these interactions were functional and of biological relevance in plants. When we compared our protein-protein interaction data with those reported by Vernoux et al. (2011), we found that 134 interaction combinations were common between studies. In contrast, we detected 79 new interactions, but failed to confirm 39 previously-reported interactions (Supplemental Table 6). The identification of 79 new interactions in the current study suggests that protein structure or regions other than CTD of ARFs likely influence ARF-Aux/IAA interactions, which was supported by ARF17, which does not contain a CTD, interacted with 9 Aux/IAA proteins including Aux/IAA5, 8, 9, 13–16, 33, and 34. Thus, using truncated ARFs might constrict the ability of ARFs to interact with Aux/IAA proteins. Consistent with this suggestion, an interaction between intact ARF1 and Aux/IAA17 was observed in our study and also previously (Ouellet et al., 2001), whereas the truncated version of ARF1 containing only CTD failed to produce positive interaction (Tiwari et al., 2003; Vernoux et al., 2011). In addition, a recent study of protein-protein interactions between ARFs and Aux/IAAs in rice indicated that full-length and truncated ARFs could differ in their capacity to interact with Aux/IAA proteins (Shen et al., 2010).

We found that all ARFs that are known to be transcriptional activators (ARF5-8, and 19), interacted with almost all Aux/IAA proteins, a result that was also found in rice (Shen et al., 2010). It is unknown whether this is the case across the plant kingdom. Consistent with previous studies, we found that ARFs that function as repressors have none-to-limited interactions with Aux/IAA proteins (Shen et al., 2010; Vernoux et al., 2011). This limited ability is not primarily due to the absence of the CTD from certain ARFs (ARF3 and 13), because other CTD-containing ARFs (ARF11 and 12) showed no interaction with Aux/IAA proteins. A recent study indicated that truncated ARFs lacking CTD can regulate gene expression in an auxin-dependent manner Wang et al. (2013), suggesting that these ARFs might function through mechanisms other than the ARF-Aux/IAA module. One such hypothesis is that ARF repressors compete with ARFs that function as activators by binding to the promoters of auxin inducible genes without forming heterodimer with Aux/IAAs (Vernoux et al., 2011). If this scenario is accurate, targeting these ARFs by Aux/IAA proteins in absence of auxin is not required. However, it seems most likely that the physical association between ARF repressors and Aux/IAA proteins is of biological significance under specific physiological or developmental circumstances, since 69 interaction combinations between 10 ARF repressors and Aux/IAA proteins were detected in yeast and some were confirmed *in planta*. In this perspective, the generation and functional characterization of double mutants of various ARF and Aux/IAA genes will deepen our knowledge about the roles of ARFs-Aux/IAA association in mediating auxin-specific responses under specific developmental and physiological settings.

While global protein-protein interaction networks provide an indication for all possible physical interaction combinations that can occur between proteins, the biological significance of such interaction requires the genes coding for these proteins to be co-expressed in particular cells. One approach to investigate the biological importance of such interactomes is to combine expression data with protein interaction data. This is because interacting proteins whose corresponding genes are co-expressed generally co-function in particular processes or pathways (Boruc et al., 2010; Klopffleisch et al., 2011). The incorporation of gene co-expression profiles with protein-protein interaction data revealed a strong correlation of gene expression for 70% of the ARF-Aux/IAA interacting pairs, providing evidence for the biological significance of these interactions. For these interacting pairs one tissue/organ was generally deduced as the site of the co-function, indicative of the specificity of the co-expression patterns. Our data point to previously unknown tissues for the majority of ARF-Aux/IAA associations and confirmed the tissue specificity of previously identified and functionally validated ARF-Aux/IAA pairs. For example, ARF7-Aux/IAA19 were found to co-function in hypocotyls (Tatematsu et al., 2004), whereas ARF7-Aux/IAA14, ARF19-Aux/IAA14, ARF7-Aux/IAA28, and ARF19-Aux/IAA28 were found to co-function in roots (Fukaki et al., 2005; De Rybel et al., 2010; Goh et al., 2012), consistent with our gene co-expression analysis. In addition, the co-expression analysis has assigned particular ARFs for specific tissue/organ. *ARF6* and *8* were found to co-express with various Aux/*IAA* genes only in flowers and floral buds. This finding is in agreement with the redundant function of ARF6 and 8 in regulating floral structures (Nagpal et al., 2005; Tabata et al., 2010).

ARF4-8 and 19, which tend to interact with all Aux/IAAs were also found to be broadly expressed and hence constitute the main hubs in the interaction and co-expression networks (Figure 5). Our data show that these ARFs have protein interactions with Aux/IAAs that can occur only in specific tissues/organs. Thus, broadly expressed *ARFs* can mediate tissue-specific functions through their association with restrictedly expressed *Aux/IAAs* such as *IAA33* and *IAA34*. It should be noted that several significant gene co-expression associations for the interacting proteins were identified in various tissues. This can be explained by the possibility that co-expression correlations may occur in various subsets of tissues or in specific cell types. In this context, analyzing the temporal and spatial gene expression patterns of the interacting protein pairs in the target tissues will precisely define their specific transcriptional signatures. On the other hand, we were unable to identify significant co-expression associations for about 30% of the interacting proteins in the tissues included in our analysis. The co-expression associations of these pairs may take place in specific tissues at particular developmental stages that are not included in our analysis. Alternatively, the co-functions of these pairs may be limited to particular physiological circumstances, including biotic and abiotic stresses.

In conclusion, our analysis confirmed most of the published protein-protein interactions between ARFs and Aux/IAAs and provided a new set of previously unknown interactions. In addition, the combination of co-expression data with protein-protein interaction data provided new leads to the site of the co-functions of ARF and Aux/IAA proteins. Taken together, these analyses set the stage for detailed functional analysis to reveal the biological significance of ARF-Aux/IAA interactions in the morphogenesis and development of various plant tissues and organs.

### Conflict of interest statement

The authors declare that the research was conducted in the absence of any commercial or financial relationships that could be construed as a potential conflict of interest.
